# The Ratio of the Hemoglobin to Red Cell Distribution Width Combined with the Ratio of Platelets to Lymphocytes Can Predict the Survival of Patients with Gastric Cancer Liver Metastasis

**DOI:** 10.1155/2021/8729869

**Published:** 2021-01-09

**Authors:** Zhao Zhai, Jialiang Gao, Ziyu Zhu, Xiliang Cong, Shenghan Lou, Bangling Han, Xin Yin, Yu Zhang, Yingwei Xue

**Affiliations:** Department of Gastroenterological Surgery, Harbin Medical University Cancer Hospital, 150 Haping Road, Harbin, Heilongjiang 150081, China

## Abstract

**Background:**

Hemoglobin/red cell distribution width (HR) and platelet/lymphocyte (PLR) ratios are considered effective prognostic markers in various cancers. We have proposed a new prognostic parameter: HR+PLR. The aim of this study is to explore the prognostic value of the HR+PLR scoring system in patients with gastric cancer liver metastasis.

**Methods:**

This study retrospectively analyzed the clinical data of 306 patients with gastric cancer liver metastases admitted to our hospital from 2007 to 2014. According to the size of HR value and PLR value, we will divide the patients into three groups, namely, HR+PLR: (1) 0 points: HR > 1.02 and PLR < 128; (2) 1 point: HR > 1.02 and PLR > 128 and HR < 1.02 and PLR < 128; and (3) 2 points: HR < 1.02 and PLR > 128.

**Results:**

The HR+PLR score was statistically different from age (*P* = 0.049), T stage (*P* < 0.001), N stage (*P* = 0.017), number of liver metastases (*P* = 0.018), gastrectomy (*P* < 0.001), hepatectomy (*P* = 0.001), peritoneal metastasis (*P* = 0.012), prognostic nutritional index (PNI) (*P* = 0.028), and neutrophil/lymphocyte ratio (NLR) (*P* = 0.045). The HR+PLR scoring system has a higher area under the ROC curve (AUC value) than PNI, PLR, HR, and PLR (AUC = 0.798, *P* < 0.001). In multivariate analysis, gastrectomy (*P* = 0.001), hepatectomy (*P* < 0.001), chemotherapy (*P* = 0.014), and HR+PLR score (*P* < 0.001) were considered independent prognostic factors.

**Conclusion:**

For patients with gastric cancer liver metastasis, the HR+PLR score is a simple, reliable, and economic prognostic marker.

## 1. Introduction

At present, although the morbidity and mortality rates have dropped significantly, gastric cancer (GC) is still an important public health burden worldwide [1]. In 2018, nearly 450,000 cases of gastric cancer were recorded, accounting for 10.6% of all cancers. At the same time, nearly 390,000 cases of gastric cancer died, accounting for 13.6% of all cancer deaths [2]. The blame is due to the fact that patients with gastric cancer are initially asymptomatic or have nonspecific symptoms, leading to more patients with distant metastases at diagnosis. Peritoneal metastasis is recognized as the most common metastatic site of gastric cancer [3]. However, the liver is also one of the common distant metastatic sites of gastric cancer, with an incidence of 5%-34% [4]. Studies have shown that liver metastasis is an important factor leading to poor prognosis of gastric cancer. Gastric cancer liver metastasis (GCLM) is always considered a systemic disease, and surgical treatment is not the best option. Although the survival benefit of gastrectomy for gastric cancer liver metastasis has been reported, whether hepatectomy can improve the prognosis of gastric cancer liver metastasis has not been confirmed [4].

At present, the prognosis of gastric cancer is usually based on the tumor stage determined by the TNM staging system. However, most patients still have the same TNM stage and different prognoses. Although some prognostic markers have been determined to be related to the poor prognosis of gastric cancer, it is difficult for them to play a role in routine clinical practice due to additional conditions in the laboratory and additional expenses. For patients with gastric cancer, it is necessary to research and develop biomarkers that are both economical and reliable to help predict patient prognosis and guide precise treatment.

Complete blood count (CBC) is a routine test for gastric cancer patients, and hemoglobin (Hb) is an important part of it. Studies have shown that low Hb levels reflect to a certain extent the decline of the host's immune response and malnutrition, which reflects the patient's low resistance to external invasion. Moreover, the low hemoglobin concentration before treatment is a predictor of poor prognosis for various cancer patients, such as gastric cancer, esophageal squamous cell carcinoma, cervical cancer, and nasopharyngeal carcinoma [5–8]. Another important CBC parameter is the distribution width of red blood cells (RDW), which is an index used to measure the heterogeneity of circulating red blood cells. RDW has been shown to be associated with poor prognosis of lung disease, cardiovascular disease, and liver disease [9–11]. Recently, some studies have found that RDW is also related to the clinicopathological characteristics and prognosis of some malignant tumors. It can reflect the patient's tumor-related inflammation, physiological reserve, and nutritional status. The high RDW value is associated with the high invasiveness of some tumors and the stage of advanced tumors, including gastric cancer, non-small-cell lung cancer, and breast cancer [12–14].

As we all know, gastric cancer is a malignant tumor associated with inflammation. Tumor-related systemic inflammatory response plays an important role in tumor progression and prognosis. A large number of studies have found that the systemic immune score (SII), neutrophil-lymphocyte ratio (NLR), platelet-lymphocyte ratio (PLR), lymphocyte-based mononuclear cell ratio (LMR), prognostic nutritional index (PNI), and others have prognostic value in a variety of cancers (including GC) [15–17].

Although the individual significance of Hb and RDW has been proven to have prognostic significance in many cancer patients, research on the ratio of the hemoglobin-to-red cell distribution width (HR) is still limited, and the significance of evaluating gastric cancer is not yet clear. And we also combined PLR for overall evaluation, which makes this study rarely reported in similar studies. Therefore, the purpose of this study was to evaluate the prognostic significance of the HR-PLR scoring system in patients with gastric cancer liver metastases.

## 2. Method

### 2.1. Study Population

This study retrospectively analyzed patients who were diagnosed with gastric adenocarcinoma by histopathology and received treatment from the Department of Gastrointestinal Surgery, Affiliated Tumor Hospital of Harbin Medical University, from January 2007 to December 2014. Patients who met any of the following criteria were excluded: (1) incomplete clinical and pathological data and follow-up data, (2) history of blood transfusion within half a year, (3) blood diseases or autoimmune diseases and other diseases that may affect the level of measured parameters, and (4) neoadjuvant chemotherapy. Finally, a total of 306 patients with gastric cancer were included. We collected the clinical data of all patients from the medical record system of the cancer hospital. The institutional ethics committee (Ethics Committee of the Cancer Hospital Affiliated to Harbin Medical University) reviewed and approved this retrospective study.

### 2.2. Evaluation of Clinical Data

We have obtained demographic and clinical pathology data. Patient-related factors include age and gender. Gastric cancer-related factors include the depth of tumor invasion (T), the number of lymph node metastases (N), the number of liver metastases, ascites (yes or no), peritoneal metastasis (yes or no), and laboratory test results. Treatment-related factors include treatment of liver metastases, methods of lymph node dissection, and postoperative adjuvant chemotherapy (yes or no). Tumor staging is based on the Eighth Edition of the American Joint Committee on Cancer/Union International for Cancer Control (AJCC/UICC) [18]. According to the Japanese Research Society for Gastric Cancer (JRSGC) [19], H1 is solitary liver metastases confined to one lobe of the liver, H2 is solitary liver metastases distributed on both sides of the liver, and H3 is multiple liver metastases distributed in the liver (two leaves). The indications for hepatectomy include primary gastric tumor resection. Liver metastasis is a solitary metastasis confined to one lobe of the liver. The diagnosis of liver metastasis relies on the results of surgical exploration and routine abdominal computed tomography/ultrasound before gastrectomy. In this study, all patients who underwent hepatectomy underwent radical gastrectomy, and D2 lymph node dissection was used as the standard lymph node dissection. Patients who did not undergo liver resection only underwent palliative gastrectomy or laparotomy. All patients receiving chemotherapy have received at least one complete chemotherapy postoperative cycle.

We measured complete blood index counts for clinical evaluation within 3 days before surgery. RDW contains RDW-CV (RDW coefficient of variation) and RDW-SD (RDW standard deviation), and these values reflect the degree of circulating red blood cell heterogeneity. Because RDW-SD can more accurately reflect the difference in red blood cell size, we use RDW-SD as the prognostic indicator for RDW for analysis. The HR value is the ratio of hemoglobin (g/dL) to the red blood cell distribution width (%), and the PLR value is the ratio of platelets (10^9^/L) to lymphocytes (10^9^/L). According to the size of HR value and PLR value, we divided patients into three groups, namely, HR+PLR: (1) 0 points: HR > 1.02 and PLR < 128; (2) 1 point: HR > 1.02 and PLR > 128 and HR < 1.02 and PLR < 128; and (3) 2 points: HR < 1.02 and PLR > 128.

### 2.3. Statistical Analysis

We use commercially available software SPSS 22.0 for statistical analysis. *P* < 0.05 is considered statistically significant. Continuous variables that conform to the logit linear hypothesis are analyzed according to receiver operating characteristic curve (ROC) analysis to determine the best cut-off value, and the area under the ROC curve (AUC value) is used to compare the prognostic value of prognostic factors. Overall survival (OS) is defined as from the date of diagnosis to the date of death or the last follow-up. The Kaplan–Meier method was used for survival analysis, and the log rank test was used to determine the difference in survival. The chi test and Student's *t*-test were used to compare the differences in the clinicopathological characteristics of the three groups. Univariate and multivariate regression analyses were performed by Cox regression analysis to determine possible independent prognostic factors.

### 2.4. Follow-Up

The patients were followed up through outpatient review and telephone calls, every 6 months in the first 1-2 years after the operation and once every 1 year in the 3-5 years after the operation, and the quality of life, recurrence, and death of the patient were recorded. The follow-up period ends in May 2019. The median follow-up time was 11 months.

## 3. Result

### 3.1. Patient Characteristics and the Best Cut-Off Value

**Figure 1:**
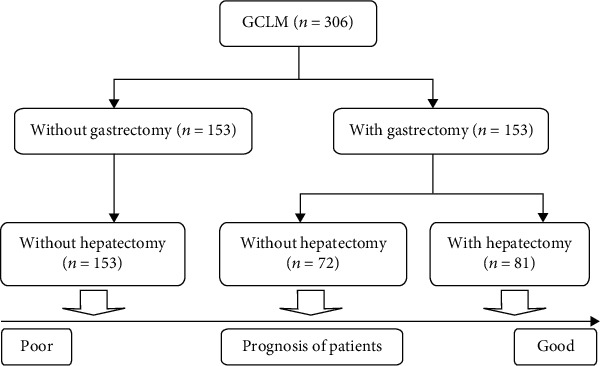
The prognostic analysis of GCLM obtained from our study first classifies patients by whether they have undergone gastrectomy and then classifies patients by whether they have undergone hepatectomy.

**Figure 2:**
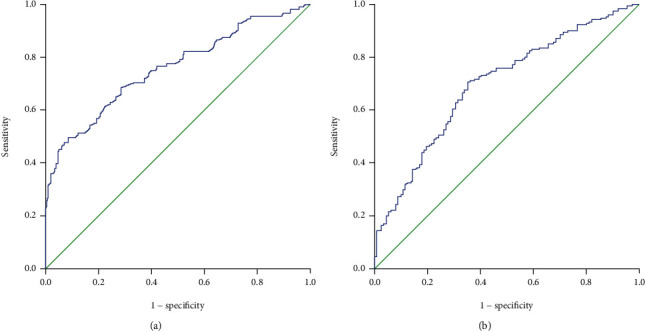
Survival ROC curve (*P* value) for HR and PLR: (a) HR (*P* < 0.001); (b) PLR (*P* < 0.001).

This study analyzed the data of 306 patients with gastric cancer liver metastases. These included 245 males (80.1%) and 61 females (19.9%), with a median age of 58 years (range 28-85 years). There were 153 and 153 patients with and without gastrectomy, respectively, and 81 and 225 patients with and without hepatectomy, respectively (see Figure 1). Their 1-year, 3-year, and 5-year survival rates were 36.2%, 12.4%, and 4.9%, respectively. In order to group and analyze patients, we used ROC curves to find the best cut-off value for laboratory results. The results showed that the best intercept value for HR was 1.02 (sensitivity 0.495, specificity 0.913, AUC = 0.757); the best intercept value for PLR was 128 (sensitivity 0.706, specificity 0.649, AUC = 0.696) (see Figure 2); the best cut-off value for NLR is 2.58 (sensitivity 0.571, specificity 0.649, AUC = 0.577); the best cut-off value for PNI is 48.1 (sensitivity 0.703, specificity 0.493, AUC = 0.572). Finally, there were 38 patients in the 0-point group, 125 in the 1-point group, and 143 in the 2-point group.

### 3.2. Relationship between the HR+PLR Score and Clinicopathological Characteristics

We explored the relationship between the HR+PLR score and the clinicopathological characteristics of patients with gastric cancer liver metastasis. As shown in Table 1, the three groups had statistical differences in age (*P* = 0.049), T stage (*P* < 0.001), N stage (*P* = 0.017), number of liver metastases (*P* = 0.018), gastrectomy (*P* < 0.001), hepatectomy (*P* = 0.001), peritoneal metastasis (*P* = 0.012), PNI (*P* = 0.028), and NLR (*P* = 0.045). There was no significant correlation in terms of gender (*P* = 0.787), chemotherapy (*P* = 0.325), and ascites (*P* = 0.127).

### 3.3. Survival Outcome and Prognostic Factors

We analyzed the impact of clinicopathological characteristics and treatment-related factors on prognosis in 306 patients with gastric cancer liver metastases. Through univariate regression analysis, we found that there are eight related factors related to the prognosis of patients with gastric cancer and liver metastasis: gastrectomy (*P* < 0.001), hepatectomy (*P* < 0.001), chemotherapy (*P* = 0.001), HR (*P* < 0.001), PLR (*P* < 0.001), PNI (*P* = 0.018), NLR (*P* = 0.017), and HR+PLR score (*P* < 0.001). The following factors have no significant effect on prognosis: gender (*P* = 0.936), age (*P* = 0.335), T stage (*P* = 0.242), N stage (*P* = 0.984), number of liver metastases (*P* = 0.440), ascites (*P* = 0.665), and peritoneal metastasis (*P* = 0.432).

When we conducted multivariate analysis to determine independent future factors, we included univariate analysis of variables with *P* < 0.05 in the Cox regression model and found that gastrectomy (*P* = 0.001), hepatectomy (*P* < 0.001), chemotherapy (*P* = 0.014), and HR+PLR score (*P* < 0.001) are independent factors affecting the prognosis of patients with gastric cancer liver metastasis (see Table 2).

**Figure 3:**
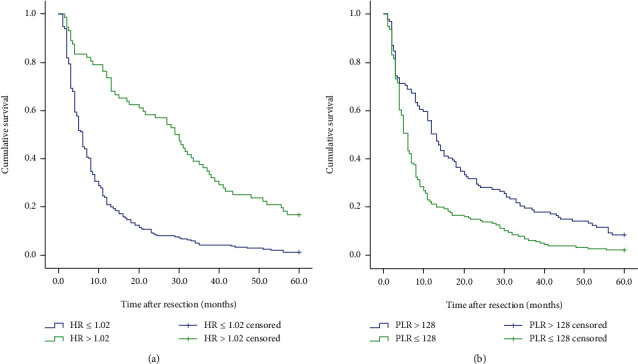
Kaplan–Meier curve for OS (overall survival) of 306 GCLM patients stratified by HR and PLR: (a) HR (*P* < 0.001); (b) PLR (*P* < 0.001).

**Figure 4:**
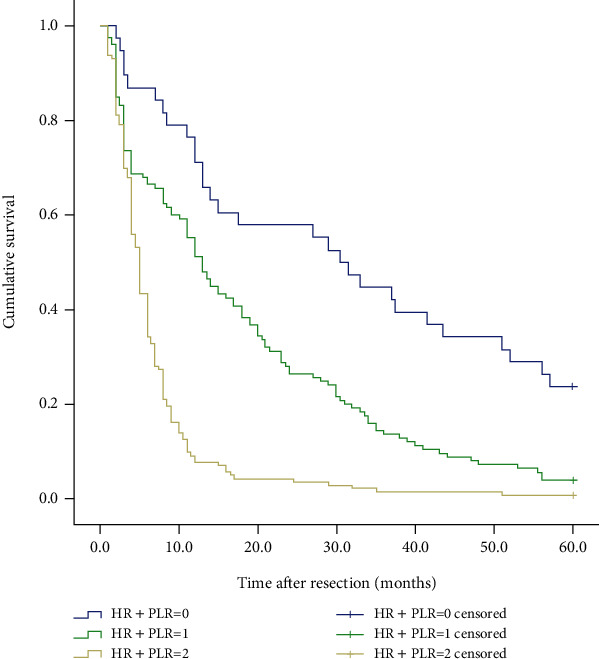
Kaplan–Meier curve for OS (overall survival) of 306 GCLM patients stratified by HR+PLR.

We also analyzed the differences in survival of 306 patients with gastric cancer liver metastases. When we separately analyze the impact of HR and PLR on the prognosis and survival of patients, as shown in Figure 3, we found that the 5-year survival rate of patients in the high HR group was significantly higher than the 5-year survival rate of patients in the low HR group, and the 5-year survival rate of patients in the low PLR group was significantly higher than the 5-year survival rate of patients in the high PLR group. The survival rate and the difference between the two groups were statistically significant (*P* < 0.001). Furthermore, we explored whether the HR+PLR scoring system can more accurately predict the prognosis of patients with gastric cancer liver metastasis. The results showed that the 5-year survival rates between the three groups were significantly different (see Figure 4). That is to say, the prognosis of patients in group 0 is significantly better than that of patients in group 1 and group 2. Therefore, we can clearly divide patients with gastric cancer liver metastasis into 3 independent prognostic groups.

### 3.4. Comparison of Prognostic Factors

**Figure 5:**
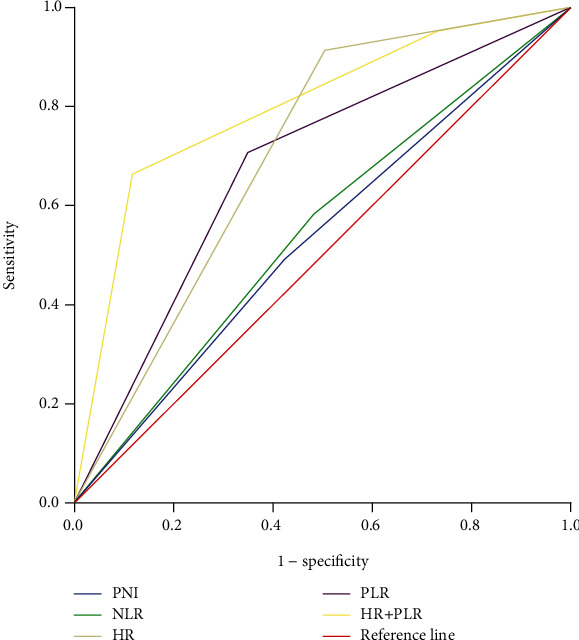
Comparison of prognostic value between the HR+PLR score and other prognostic factors.

In order to further seek the best prognostic marker for predicting postoperative survival of patients with gastric cancer liver metastasis, ROC analysis was used to compare AUC values. The results show that the HR+PLR scoring system has higher AUC values (AUC = 0.798, *P* < 0.001) than PNI, PLR, HR, and PLR (see Figure 5 and Table 3).

### 3.5. Subgroup Analysis

**Figure 6:**
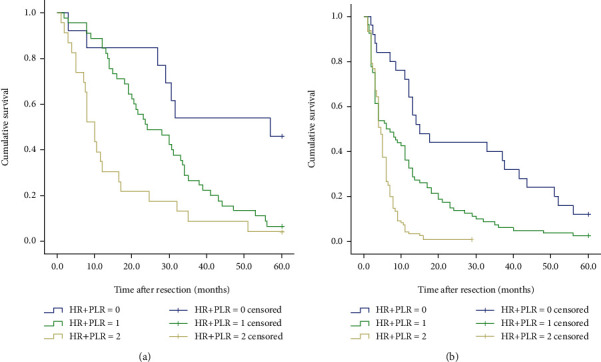
Kaplan–Meier curve for OS (overall survival) of GCLM patients stratified by HR+PLR: (a) 81 GCLM patients undergoing hepatectomy (*P* < 0.001); (b) 225 GCLM patients without hepatectomy (*P* < 0.001).

**Figure 7:**
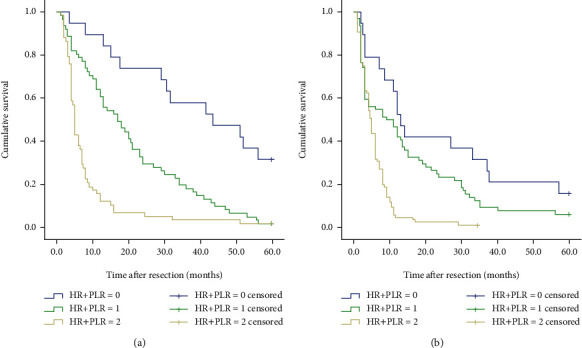
Kaplan–Meier curve for OS (overall survival) of GCLM patients stratified by HR+PLR: (a) 138 GCLM patients undergoing postoperative chemotherapy (*P* < 0.001); (b) 168 GCLM patients without postoperative chemotherapy (*P* < 0.001).

To determine whether the HR+PLR scoring system still has predictive value in patients whether they have undergone hepatectomy and whether they have undergone chemotherapy, we performed a subgroup analysis. The results showed that when hepatectomy was performed or not, the 5-year survival rates of the three groups of patients were significantly different (all *P* < 0.001) (see Figure 6). And the HR+PLR scores were independent prognostic factors for patients undergoing hepatectomy and not undergoing liver resection (see Table 4). Regardless of whether patients undergo postoperative chemotherapy, the prognosis of patients in group 2 is worse than that in group 0 or group 1 (all *P* < 0.001) (see Figure 7).

## 4. Discussion

Gastric cancer is one of the cancers with the highest invasiveness and the highest tumor burden, which causes distant metastases to be common among patients with gastric cancer. Blood metastasis is the main method of metastasis in patients with gastric cancer. Due to the abundant blood supply to the liver, the portal vein receives most of the venous return of the gastrointestinal tract, resulting in gastric cancer cells that are easily transferred to the liver through the blood, and liver metastasis also accounts for 77.8% of all blood metastases [20]. This makes the survival time and long-term survival rate of patients with gastric cancer liver metastasis always low. The short-term and long-term survival of gastric cancer patients is affected by a variety of factors. Some of the factors that have been identified include tumor stage, histological type, and degree of differentiation. But I think we need to explore some economic and practical prognostic markers that can be used in clinical practice to guide treatment and predict survival [21]. Recently, with the accumulation of evidence, a large number of studies have confirmed that some systemic inflammatory response serum parameters are potential predictors of the prognosis of various cancers [5, 12, 22, 23]. It has been reported that Hb and RDW, as part of a complete blood count, reflect the patient's nutritional status, tolerance capacity, and the size of red blood cell heterogeneity [13, 24]. High PLR is considered to be an influencing factor for poor prognosis in various cancers [25, 26]. Although the prognosis of gastric cancer by conventional blood test markers is still controversial, this study found that there are significant differences in the 1-year, 3-year, and 5-year survival rates among the three groups of patients with gastric cancer liver metastases using the HR+PLR score. Therefore, combining HR and PLR as a stratified study to enrich the prognosis of gastric cancer patients and a scoring system to predict prognosis is reasonable and feasible.

Anemia is extremely common in cancer patients, accounting for about 30% [24]. For example, in gastric cancer, lung cancer, prostate cancer, and ovarian cancer, preoperative low hemoglobin concentration is a negative factor for postoperative complications and prognosis of patients [12, 24, 27]. Hypoxia caused by anemia overexpresses hypoxia-inducible factor-1 (HIF-1), thereby inducing vascular endothelial growth factor, glucose transporter, epidermal growth factor, and glycolytic enzyme to promote tumor metabolism [28]. In addition, the acceleration of tumor blood vessel growth caused by hypoxia can increase the resistance of tumor cells to perioperative radiotherapy and chemotherapy, resulting in poor survival [29].

RDW as a parameter representing the size of circulating red blood cell heterogeneity, in essence, reflects the type of anemia and nutritional status of patients. The role of RDW is getting more and more attention. Prior to this, studies have confirmed that RDW can accurately predict the activity of inflammatory bowel disease, the inflammatory state of hepatitis B virus patients, and the mortality of acute pancreatitis [11, 30, 31]. Other studies have found that RDW is closely related to the risk of cardiovascular disease [32]. Recent studies have found that high RDW values can lead to increased risk and poor prognosis of various malignant tumors, including gastric cancer, multiple myeloma, lung cancer, and esophageal cancer [5, 33, 34]. The study by Hirahara et al. showed that RDW is considered to be an independent prognostic factor for OS (overall survival) and CSS (cancer-specific survival) in elderly and nonelderly patients [35]. In another study, Yazici et al. conducted a study of 172 patients undergoing radical gastrectomy and found that patients with high RDW values had a higher incidence of advanced gastric cancer and high RDW values were strongly associated with short-term mortality [13]. These evidences support the significant correlation between high RDW value and poor prognosis, but the association mechanism between RDW and survival of gastric cancer patients is not clear. Recent studies have found that RDW is closely related to tumor-related inflammation and nutritional status. Under the action of a variety of inflammatory cytokines, it inhibits the stimulation and maturation of erythropoietin on bone marrow erythroid stem cells, resulting in an increase in immature red blood cells and increased heterogeneity in the peripheral blood circulation [35]. In addition, the tumor microenvironment plays an important role in the occurrence, development, and metastasis of tumors, which accelerates tumor progression.

Although a large number of studies have reported the separate effects of Hb and RDW on the prognostic value of gastric cancer, studies have confirmed the predictive power of Hb/RDW in lung cancer, esophageal cancer, and head and neck cancer [5, 12], but Hb/RDW research on the prognostic impact of gastric cancer patients is still limited. As we all know, Hb and RDW are recognized as effective nutritional indicators and potential prognostic factors in gastric cancer. However, both Hb and RDW are susceptible to various non-tumor-related factors, so Hb/RDW can minimize any potential risk of bias. According to reports, research by Yılmaz et al. found that low NLR, low SII, and high HR are associated with longer DFS (disease-free survival) and OS (overall survival). In univariate analysis, NLR, SII, and HR are significantly correlated with prognosis. However, in multivariate analysis, only HR is considered to be an independent prognostic factor for DFS/OS [36]. Our study also supports this view and found that the 5-year survival rate of patients in the high HR group was significantly higher than that in the low HR group (*P* < 0.001).

The impact of HR on the prognosis of patients with gastric cancer liver metastasis is not only related to the related inflammatory response but also restricted by the physical health of the patients at that time. Because HR is susceptible to complex tumors or non-tumor-related factors, we believe that HR indicators alone cannot give rigorous and accurate prognostic information. There have been a large number of reports before combining various inflammatory indicators to establish several inflammatory scoring systems, for example, the RDW+NLR score [37] and fibrinogen+NLR (F-NLR) score [38]. Since NLR has been widely studied as an indicator of systemic inflammation [39], so for the first time, we propose a new scoring system: HR+PLR, which is used to predict the prognosis of patients with gastric cancer and liver metastases. The results of this study found that HR+PLR is an independent prognostic factor for 5-year survival, and the 5-year survival rates of group 0, group 1, and group 2 have significant differences (*P* < 0.001). We also analyzed the association between different HR+PLR groups and clinicopathological characteristics. The proportion of T4b patients in group 2 was significantly higher than that in group 0 and group 1, and the proportion of patients with multiple liver metastases in group 2 was significantly higher. These evidences show that low HR and high PLR may be indicators of aggressive tumor behavior.

Liver metastases are fatal to gastric cancer patients and have always been an important factor in the death of gastric cancer patients. However, a standardized treatment system for patients with gastric cancer liver metastases has not been established. Liver metastases of gastric cancer are usually distributed in two liver lobes. Most patients present with H2 or H3 metastases. However, we currently consider patients with solitary liver metastases or multiple metastases limited to one liver lobe suitable for and benefiting from hepatectomy. This has led to only a few patients suitable for liver resection. Therefore, we conducted a subgroup analysis to explore the prognostic relationship between different groups in patients undergoing hepatectomy and patients who did not undergo hepatectomy. The results showed that the 5-year survival rates of the three groups of patients were significantly different (all *P* < 0.001) (see Figure 5). This also proves the reliability of the HR+PLR score.

In order to eliminate other potential risks of bias, we also added the well-known inflammation parameters PNI and NLR to the Cox regression model. Multivariate analysis showed that only the HR+PLR score was an independent prognostic factor. And the AUC value of the HR+PLR score is 0.798, which is significantly better than PNI, NLR, HR, and PLR. This indicates that the scoring system is superior to other inflammatory markers in predicting the prognosis of patients with gastric cancer liver metastases. HR and PLR can be quickly and easily calculated by CBC, and there is no additional cost. Therefore, the HR+PLR score can be used as a simple, reliable, and economical predictive marker to help patients with gastric cancer and liver metastasis.

This study still has certain limitations that are worth discussing. (1) The main limitation of this study is its retrospective design, which prevents us from completely excluding all influencing factors in the research process, and HR and PLR are extremely susceptible to systemic inflammatory diseases. (2) This study is a single-center study that only included a small number of patients, which led us to only perform limited statistics and inferences. Therefore, this result also needs to be verified using larger patient queues and multicenter data. (3) Due to the long time span of the study, this article failed to fully analyze the effects of chemotherapy regimens, periodicity, and postoperative complications on the prognosis of patients after surgery. (4) When we grouped patients according to whether they had undergone gastrectomy, we failed to continue to subdivide patients undergoing radical gastrectomy into hepatectomy and no hepatectomy, resulting in heterogeneous results.

## 5. Conclusion

This study first proposed the prognostic value of the HR+PLR scoring system for patients with gastric cancer liver metastases. It was found that there is a significant correlation between the HR+PLR scoring system and the clinicopathological characteristics and survival results of patients with gastric cancer liver metastasis. In multivariate analysis, the HR+PLR scoring system is also an independent prognostic factor. Therefore, the HR+PLR score can be used as a reliable, simple, and economic prognostic marker in the clinical practice of gastric cancer liver metastases.

## Figures and Tables

**Table 1:** Comparison of clinicopathological characteristics of different HR+PLR groups.

Variables	HR+PLR = 0, *n* (%)	HR+PLR = 1, *n* (%)	HR+PLR = 2, *n* (%)	*P*
Age				0.049
<60	28 (73.6)	65 (52)	76 (53.1)	
≥60	10 (26.4)	60 (48)	67 (46.9)	
Gender				0.787
Male	32 (84.2)	100 (80)	113 (79)	
Female	6 (15.8)	25 (20)	30 (21)	
T stage				<0.001
T4b	10 (26.4)	64 (51.2)	88 (61.5)	
Not T4b	20 (52.6)	61 (48.8)	55 (38.5)	
N stage				0.017
N0 or N1 or N2	19 (50)	41 (32.8)	50 (35)	
N3a or N3b	19 (50)	84 (67.2)	93 (65)	
Number of liver metastases				0.018
Single	15 (39.5)	65 (52)	50 (35)	
Multiple	23 (60.5)	60 (48)	93 (65)	
Gastrectomy				<0.001
Yes	29 (76.3)	74 (59.2)	50 (35)	
No	9 (23.7)	51 (40.8)	93 (35)	
Hepatectomy				0.001
Yes	13 (34.3)	45 (36)	23 (16.1)	
No	25 (65.7)	80 (64)	120 (83.9)	
Chemotherapy				0.325
Yes	19 (50)	61 (48.8)	58 (40.6)	
No or unknown	19 (50)	64 (51.2)	85 (59.4)	
Ascites				0.127
Yes	12 (31.6)	21 (16.8)	33 (23.1)	
No	26 (68.4)	104 (83.2)	110 (76.9)	
Peritoneal metastasis				0.012
Yes	16 (34.3)	21 (16.8)	32 (22.4)	
No	25 (65.7)	104 (83.2)	111 (77.6)	
PNI				0.028
≤48.1	15 (39.5)	50 (40)	78 (54.5)	
>48.1	25 (60.5)	75 (60)	65 (45.5)	
NLR				0.045
≤2.58	18 (47.4)	66 (52.8)	54 (37.8)	
>2.58	20 (52.6)	59 (47.2)	89 (62.2)	

HR: hemoglobin-to-red cell distribution width; PLR: platelet-lymphocyte ratio; PNI: prognostic nutritional index; NLR: neutrophil-lymphocyte ratio.

**Table 2:** Univariate and multivariate analyses of total survival parameters.

Variables	Univariable			Multivariable		
	HR	95% CI	*P*	HR	95% CI	*P*
Gender	1.012	0.758-1.350	0.936			
Age	1.121	0.889-1.412	0.335			
T stage	0.869	0.698-1.099	0.242			
N stage	0.998	0.790-1.259	0.984			
Number of liver metastases	1.096	0.868-1.384	0.440			
Gastrectomy	2.858	2.237-3.651	<0.001	1.651	1.218-2.236	0.001
Hepatectomy	2.566	1.950-3.377	<0.001	1.996	1.419-2.806	<0.001
Chemotherapy	1.469	1.164-1.854	0.001	1.343	0.063-1.697	0.014
Ascites	1.065	0.802-1.413	0.665			
Peritoneal metastasis	1.118	0.847-1.476	0.432			
HR	0.328	0.244-0.442	<0.001			
PLR	1.755	1.382-2.230	<0.001			
PNI	0.755	0.599-0.953	0.018	0.814	0.639-1.037	0.096
NLR	1.326	1.051-1.673	0.017	0.993	0.778-1.266	0.993
HR+PLR	2.348	1.934-2.850	<0.001	2.149	1.751-2.638	<0.001

HR: hazard ratio; 95% CI: 95% confidence interval.

**Table 3:** Comparison of prognostic value between the HR+PLR score and other prognostic factors.

Prognostic score	Area under the ROC curve (95% CI)	*P* value
PNI	0.534 (0.467-0.602)	0.316
NLR	0.549 (0.482-0.616)	0.145
PLR	0.678 (0.615-0.742)	<0.001
HR	0.704 (0.639-0.769)	<0.001
HR+PLR	0.798 (0.747-0.849)	<0.001

95% CI: 95% confidence interval.

**Table 4:** Univariate and multivariate analyses of GCLM patients with and without hepatectomy.

Variables	Patients undergoing hepatectomy						Patients without hepatectomy					
	Univariable			Multivariable			Univariable			Multivariable		
	HR	95% CI	*P*	HR	95% CI	*P*	HR	95% CI	*P*	HR	95% CI	*P*
Gender	0.684	0.386-1.213	0.194				0.356	0.969-1.896	0.085			
Age	0.858	0.537-1.369	0.520				1.462	1.117-1.913	0.006	1.225	0.933-1.609	0.144
T stage	1.012	0.635-1.613	0.961				0.899	0.684-1.182	0.446			
N stage	0.586	0.365-0.942	0.027	0.641	0.389-1.057	0.082	1.071	0.816-1.408	0.620			
Number of liver metastases	0.847	0.528-1.359	0.491				1.016	0.774-1.335	0.906			
Chemotherapy	1.662	1.040-2.657	0.034	1.635	1.007-2.654	0.047	1.323	1.012-1.731	0.041	1.283	0.978-1.682	0.072
Ascites	1.606	0.821-3.144	0.167				1.064	0.777-1.458	0.698			
Peritoneal metastasis	0.172	0.641-2.114	0.606				1.255	0.916-1.719	0.157			
HR	0.334	0.191-0.584	<0.001				0.288	0.199-0.417	<0.001			
PLR	1.423	0.890-2.275	0.140				1.644	1.235-2.188	0.001			
PNI	0.539	0.333-0.871	0.012	0.794	0.467-1.352	0.396	0.828	0.635-1.080	0.164			
NLR	1.209	0.756-1.933	0.428				1.221	0.933-1.599	0.146			
HR+PLR	2.288	1.566-3.344	<0.001	1.933	1.246-2.998	0.003	2.215	1.757-2.793	<0.001	2.143	1.698-2.705	<0.001

HR: hazard ratio; 95% CI: 95% confidence interval.

## Data Availability

The datasets used and analyzed in the present study are available from the corresponding author upon reasonable request.
